# The association between subclinical hypothyroidism and TPOAb positivity with infertility in a population-based study: Tehran thyroid study (TTS)

**DOI:** 10.1186/s12902-021-00773-y

**Published:** 2021-05-26

**Authors:** Batul Birjandi, Fahimeh Ramezani Tehrani, Atieh Amouzegar, Maryam Tohidi, Razieh Bidhendi Yarandi, Feriedoun Azizi

**Affiliations:** 1 Endocrine Research Center, Research Institute for Endocrine Sciences, Shahid Beheshti University of Medical Sciences, P.O. Box: 19395-4763, Tehran, I.R Iran; 2 Reproductive Endocrinology Research Center, Research Institute for Endocrine Sciences, Shahid Beheshti University of Medical Sciences, Tehran, Iran; 3 Prevention of Metabolic Disorders Research Center, Research Institute for Endocrine Sciences, Shahid Beheshti University, Tehran, Iran; 4 Department of Biostatistics, University of Social Welfare and Rehabilitation Sciences, Tehran, Iran

**Keywords:** Thyroid TPO positivity, Infertility

## Abstract

**Background:**

Thyroid autoimmunity(TAI) is the most prevalent autoimmune condition in women of fertile age. There are increasing data regarding the association of thyroid dysfunction and thyroid autoimmunity with adverse pregnancy outcomes but there is no consensus regarding infertility and TPOAb positivity; thus we aimed to evaluate the association between thyroid TPOAb positivity and infertility in females and males in a population-based study (TTS).

**Methods:**

Cross-sectional study of 3197 female and male participants in Tehran Thyroid Study (TTS) at the framework of the Tehran Lipid and Glucose Study (TLGS). Data included biochemical measurements and a self-administered questionnaire.

**Results:**

A total of 12,823 cases in phase 4, 3719 cases (2108 female and 1611 male) were analyzed. The mean TSH of the infertile female and male was 2.52 ± 2.68 μIU/ml and 3.24 ± 10.26 μIU/ml respectively. The TPO median(IQR) of women with and without a history of infertility were 6.05 (3.30–13.96)and 6.04 (3.17–11.15);(*P* = 0.613), they were 5.08 (3.20–125.68) and 5.31 (3.93–125.68);(*P* = 0.490) in male participants, respectively. Results of crude and adjusted logistic regression analysis of the development of infertility by thyroid function and TPOAb, except for fT4 in male subjects, depicted no association between infertility and other variables in both crude and adjusted models.

**Conclusion:**

Based on the result, thyroid autoimmunity was not associated with infertility in both females and males.

**Supplementary Information:**

The online version contains supplementary material available at 10.1186/s12902-021-00773-y.

## Background

Thyroid autoimmunity (TAI) is defined presence of cell and humoral immune response against thyroid antigens with subsequent infiltration of T cells and B cells, autoantibodies production and, finally, the development of different clinical manifestations [1]. TAI is the most prevalent autoimmune condition in women of fertile age [2]. The mechanisms describe including thyroid-stimulating hormone (TSH) dependent mechanism or independent of TSH in a variety of mechanisms such as immunoregulatory, vitamin D deficiency and cross-reactivity of thyroid autoantibodies to extra thyroid sites [3]. There is increasing data regarding the association of thyroid dysfunction and thyroid autoimmunity with adverse pregnancy outcomes such as recurrent miscarriage and recurrent embryo implantation failure [4–6].

Although the uterus and ovary in females and gonadal steroids, testosterone level,spermatogenic function in males can be targeted by thyroid antibodies, there is no consensus regarding the exact mechanism of infertility influenced by thyroid autoimmunity [7, 8]. The results of available studies on the association between TAI and infertility are rare and controversial especially in males and are limited by study designs, small sample size, heterogeneity in participants and lack of complete sociodemographic data [9–12]. Additionally, there are few population-based studies to reveal this association [13].

In the Iranian population, the prevalence of primary infertility is 17.3% in couples indicates that is a common problem in our society [14].; thus we aimed to evaluate.

the association between thyroid peroxidase antibody (TPOAb) positivity and infertility in a population-based study in both male and female.

## Methods

### Participants

Details of the present study design have been reported elsewhere [15]. In short, this is a cross-sectional study within the framework of the Tehran Thyroid Study (TTS), a prospective population-based cohort study was conducted on residents of Tehran’s 13th district between March 1997 and December 2004. A total of 5769 participants aged ≥20 years, were selected using multistage cluster random sampling. The TTS is being conducted within the framework of Tehran Lipid and Glucose Study (TLGS), an ongoing community base study with 3-year interval follow-ups, for identification and prevention of non- communicable disease.

For the purpose of the present study, we excluded all those unmarried women, those with unwilling pregnancy and duration of marriage below 1 year. A specific questionnaire including age, education, reproductive history, contraceptive behaviors, smoking, menstrual pattern, fertility history (including age of marriage, number of parity, number of birth, time to pregnancy, number of abortion) and seeking medication for infertility was collected through face to face interviews by a trained midwife. For those ones with uncertain data in terms of infertility, medical documents had been evaluated (supplementary data). The content validity of this questionnaire was assessed by 15 gynecologists and reproductive health experts. The reliability of the instrument was determined using test-retest to determine the level of agreement between responses in a 10 days-interval (*r* = 0.91). Internal consistency was measured using Cronbach’s alpha correlation coefficient (α = 0.79) details have been published elsewhere [16].

Male infertility was identified based on evaluating the medical documents of the infertile couples.

**Fig. 1:**
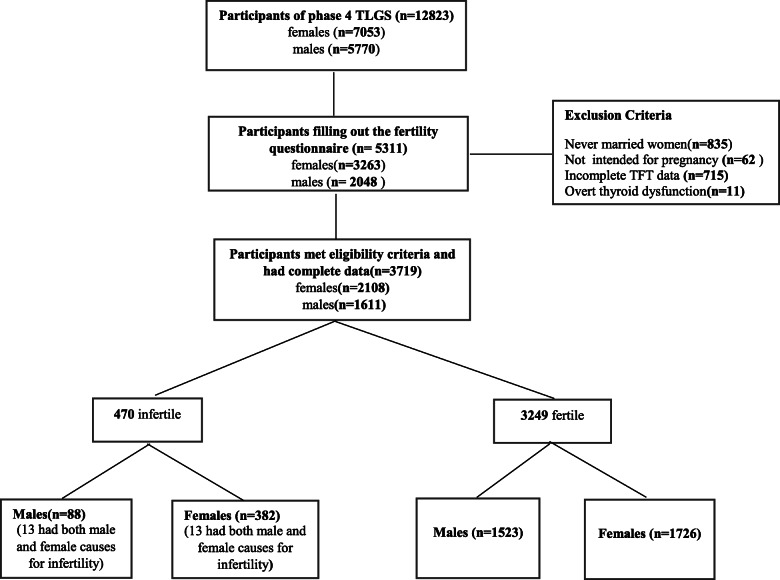
Study flowchart. TLGS, Tehran Lipid and Glucose Study; TFT, Thyroid function test

Out of 12,823 subjects participated in 3rd follow-up of TLGS, we excluded all those never married women or men (*n* = 835), those couples who had never willingness for having a baby (*n* = 62), those with uncertain data regarding reproductive and fertility history (*n* = 3969) and those with missing values of TSH, free T4 (fT4), TPOAb (*n* = 715), those overt Hypothyroidism/ Hyperthyroidism(*n* = 11). Finally, there were 3629 participants including 2108 females (382 with infertility) and 1611 males (88 with infertility) who met our eligibility criteria for the purpose of the present study. A total of 470 infertile (88 males and 382 females) and 3249 fertile (1523 males and 1726 females). The study flowchart is presented in Fig. 1.

### Definition terms

- infertility was defined as the failure to achieve a clinical pregnancy after 12 months or more of regular unprotected sexual intercourse whether or not having a child due to male or female factors [17].
- Number of birth: the number of complete expulsion or extraction from a woman of a fetus after 22 completed weeks of gestational age, irrespective of whether it is a live birth or stillbirth [17].
- Number of abortion was defined the number of pregnancy loss without outside intervention before 20 weeks gestation [18].
- Time to pregnancy was defined the time between the decision to pregnancy and become pregnant) measured in months or in numbers of menstrual cycles [17].
- Thyroid status has been subcategorized as follows; Euthyroidism was defined as normal TSH within the range of 0.32–5.06 mIU/L and fT4 of 0.91–1.55 ng/dL. Subclinical hypothyroidism was defined as TSH levels > 5.06 and < 10 mIU/L and fT4 of 0.91–1.55 ng/dL and not using LT4. Subclinical hyperthyroidism was defined as TSH levels < 0.32 mIU/L and fT4 of 0.91–1.55 ng/dL. TPOAb positivity was defined as TPOAb> 35 IU/mL for females and TPOAb> 32 IU/mL for males [19].

### Laboratory measurements

All laboratory assays were performed on serum samples stored at − 70 °C. TSH and fT4 were measured by the electrochemiluminescence immunoassay (ECLIA), using Roche Diagnostics kits & Roche/Hitachi Cobas e-411 analyzer (GmbH, Mannheim, Germany). For monitoring accuracy of assays, lyophilized quality control material (Lyphochek Immunoassay Plus Control; Bio-Rad Laboratories, Irvine, CA, USA) was used; intra- and inter-assay coefficients of variation (CV) were 1.3 and 3.7% for fT4 and 1.5 and 4.5% for TSH measurements, respectively. TPOAb was determined by immunoenzymometric assay (IEMA) using commercial kits (Monobind, Costa Mesa, CA, USA) and the Sunrise reader (Tecan Co., Salzburg, Austria); intra- and inter-assay CVs were 3.9 and 4.7%, respectively. All immunoassay tests were performed in the same laboratory by skilled laboratory technicians.

The study was approved by the Regional Ethics Commottee of Shahid Beheshti, Iran (Reg.number IR.SBMU.MSP.REC.1399.282) and comfirmed to the principle of the Declaration of Helsinki.

The flowchart of participants shows in Fig. 1.

### Statistical analysis

Descriptive statistics were presented in mean(±SD) or median (IQR) for normal and non-normal varibles and n(%) for categorical data. For two groups and three groups comparisions, t-test or mann-whitney U tests and ANOVA or Kruskal Wallis tests for normal and non-normal continuous variables was applied. Crude and adjusted Logistic regression were used to estimate odds ratio (95% CI) of infertility. Crude and adjusted linear regression models were used to model the time to pregnancy. Statistical analysis was done using STATA software (version 10; STATA, INC, college station, TX, USA).

## Results

Of all 2108 enrolled women, 382 women had a history of female infertility. In male participants, there were 88 men with a history of infertility out of 1611 ones. Table 1 presents the characteristics of study participants according to fertility history. The mean age (±SD) of women with and without a history of infertility were 39y (±7)and 40y (±7) in the fertile group (*P* = 0.02), they were 59y(±14) and 54y(±11) in fertile and infertile male participants, respectively. The median(IQR) of TPO-Ab in women with and without a history of infertility were 6.04 (3.17–11.15) and 6.05 (3.30–13.96) IU/ml;(*P* = 0.613), they were 5.31 (3.93–125.68) and 5.08 (3.20–125.68) IU/ml;(*P* = 0.490) in male participants respectively .

**Table 1:** baseline characteristic in participants.if normal distribution mean if not median

	Hx of Fertility	Hx of Infertility	* ***P***value
^**a**^**Female**			
**Age (Y)**	**40(±7)**	**39(±7)**	* 0.021
**TTP (mth)**	**2.00 (1.00–4.00)**	**15.00 (12.00–36.00)**	* 0.000
**No of birth**	**2.00 (2.00–3.00)**	**2.00 (1.00–3.00)**	* 0.001
**No of parity**	**2.00 (2.00–3.00)**	**2.00 (1.00–3.00)**	* 0.001
**No of abortion**	**0.00 (0.00–1.00)**	**0.00 (0.00–1.00)**	**0.452**
**BMI (kg/m**^**2**^**)**	**28.26 (25.33–31.25)**	**28.44 (25.43–32.44)**	**0.241**
**SBP (mmHg)**	**109.34(±12.76)**	**109.45(±14.05)**	**0.601**
**DBP (mmHg)**	**73.53(±9.88)**	**73.87(±10.48)**	**0.617**
**FBS (mg/dl)**	**87.00 (81.00–93.00)**	**88.00 (82.00–94.00)**	**0.193**
**FT4(ng/dl)**	**1.18 (1.08–1.30)**	**1.18 (1.08–1.32)**	**0.724**
**TSH (mU/L)**	**1.83 (1.07–3.06)**	**2.04 (1.11–3.31)**	**0.165**
**TPOAb (IU/ml)**	**6.05 (3.30–13.96)**	**6.04 (3.17–11.15)**	**0.613**
^**a**^**Male**			
**Age(Y)**	**54(±11)**	**59(±14)**	*0.003
**BMI (kg/m2)**	**26.89 (24.49–34.21)**	**25.69 (23.88–37.33)**	**0.190**
**SBP (mmHg)**	**119.11(±17.45)**	**127.12(±24.16)**	* 0.000
**DBP (mmHg)**	**78.21(±10.73)**	**80.48(±12.86)**	**0.155**
**FBS (mg/dl)**	**92.00 (86.00–141.00)**	**94.00 (88.00–205.00)**	* 0.011
**TPOAb (IU/ml)**	**5.08 (3.20–125.68)**	**5.31 (3.93–125.68)**	**0.490**
**FT4(ng/dl)**	**1.30 (1.16–1.63)**	**1.26 (1.11–1.57)**	* 0.042
**TSH (mU/L)**	**1.50 (0.87–6.17)**	**1.79 (0.89–7.21)**	**0.196**

^a^Data are shown as mean ± SD and median, IQR. ‡*IQR* interquartile range, *Y* year, *TTP* Time to pregnancy was defined just for participants with Hx of pregnancy (the time between the decision to pregnancy and become pregnant), *mth* month, *BMI* body mass index, *SBP* systolic blood pressure, *DBP* diastolic blood pressure, *FBS* fasting blood sugar, *TSH* thyrotropin, *FT4* free thyroxine, *TPO* thyroid peroxidase antibody

*P-value obtained from t-student or Mann-Whitney U tests for normal or non-normal continuous variables,significant level was considered as *p*-value < 0.05

Table 2 presents the Odds Ratio and 95% Confidence interval of TPOAb positivity and female infertility and abortion; it was 0.98 95%CI: (0.7–1.4); *P* = 0.721 for infertility and 1.04 95%CI: (0.8–1.4)); *P* = 0.845, respectively.

**Table 2:** The Odds Ratio and 95% Confidence interval of TPOAb status and female infertility, abortion, thyroid dysfunction with controls

	TPO antibody		*OR (95%CI)	*p*-value
	Positive (≥35)	Negative (< 35)		
**Infertility**				
Yes	50 (17.9%)	332 (18.2%)	0.98 (0.7–1.4)	0.721
No	230 (82.1%)	1496 (81.8%)	REF	
**Abortion**				
Yes	92 (32.9%)	585 (32.0%)	1.04 (0.8–1.4)	0.845
No	188 (67.1%)	1243 (68.0%)	REF	

* significant *p*-value< 0.05

^Ψ^ logistic regression was applied to obtain crude OR (95%CI)

The result of crude and adjusted logistic regression analysis of thyroid function tests and infertility (both male and female) is presented in Table 3. It has been shown that except for fT4 in male participants, there was no association between infertility and other thyroid function tests in both crude and adjusted models (Table 3; it has been shown that the odds ratio of male infertility was increased by 0.189 95% CI (0.056, 0.637), with each unit increase in Ft4; the results remained statistically significant after multiple adjustment by Age, education, BMI, Smoking, Systolic&Diastolic BP, FBS (0.26595% CI (0.075, 0.940), *p* = 0.040. Linear regression analysis revealed no association between time to pregnancy, the number of abortions and parity with TSH,fT4 and TPOAb positivity in women (Table 4); moreover we found no association between infertility (both male and female) with TSH,fT4 and TPOAb positivity (Table 4).

**Table 3:** Results of crude and adjusted logistic regression analysis of infertility by thyroid function and TPOAb status

Female			Male		
**Crude models**					
		*OR of infertility (95%CI)	***P*_value**	*OR of infertility (95%CI)	***P*_value**
FT4 (ng/dl)		0.997 (0.602–1.650)	0.991	0.189 (0.056, 0.637)	* 0.007
TSH (mU/L)		1.015 (0.976–1.054)	0.458	1.028 (1.001, 1.055)	* 0.042
TPOAb (IU/ml)		0.999 (0.998–1.000)	0.222	1.00 (0.996, 1.003)	0.833
TPOAb	Positive	1.0 (0.8,1.35)	0.953	0.7 (0.3,1. 5)	0.392
	Negative	(ref)		(ref)	
**Age-and-BMI-Adjusted models**					
FT4 (ng/dl)		1.003 (0.600–1.676)	0.991	0.280 (0.081, 0,967)	* 0.044
TSH (mU/L)		1.012 (0.974–1.052)	0.536	1.025 (0.998, 1.052)	0.070
TPOAb (IU/ml)		0.999 (0. 998–1.000)	0.194	1.00 (0.996, 1.003)	0.874
TPOAb	Positive	0.97 (0.7,1.35)	0.888	0.7 (0.3,1.6)	0.416
	Negative	(ref)		(ref)	
^**Ψ**^**Multi-Adjusted models**					
FT4 (ng/dl)		1.007 (0.596–1.702)	0.978	0.265 (0.075, 0.940)	* 0.040
TSH (mU/L)		1.012 (0.973–1.052)	0.552	1.026 (0.999, 1.053)	0.061
TPOAb (IU/ml)		0.999 (0.998–1.000)	0.196	1.00 (0. 997, 1.003)	0.915
TPOAb	Positive	0.983 (0.694–1.393)	0.924	0.778 (0.304,1.992)	0.610
	Negative	(ref)		(ref)	

*significant *p*-value < 0.05

^Ψ^Adjusted by Age, education, BMI, Smoking, Systolic Blood Pressure (SBP), Diastolic Blood Pressure (DBP), Fasting blood Sugar (FBS)

**Table 4:** Results of crude and adjusted linear regression analysis for the time to pregnancy, number of abortions & parity by thyroid function and TPOAb status

Exposure (time to pregnancy)		Coefficient (95%CI)	* ***P***_value
**Crude models**			
FT4 (ng/dl)		−0.890 (−4.168–2.388)	−0.594
TSH (mU/L)		0.086 (−0.167–0.340)	0.505
TPOAb (IU/ml)		−0.00 (− 0.006–0.006)	0.891
TPOAb	Positive	0.64 (−2.1, 3.4) 0.648	
	Negative	(ref)	
**Adjusted model 1#**			
FT4 (ng/dl)		−0.768 (−4.118–2.592)	0.644
TSH (mU/L)		0.079 (−0.175–0.333)	0.543
TPOAb (IU/ml)		−0.00 (− 0.006–0.006)	0.958
TPOAb	Positive	0.56 (−2.1, 3.3)	0.688
	Negative	(ref)	
**Adjusted model 2##**			
FT4 (ng/dl)		−0.759 (−4.109–2.592)	0.657
TSH (mU/L)		0.087 (−0.168–0.343)	0.502
TPOAb (IU/ml)		−0.00 (− 0.006–0.006)	0.986
TPOAb	Positive	0.56 (−2.1, 3.3)	0.688
	Negative	(ref)	
**Exposure number of Abortion**		**The coefficient (95%CI)**	***P_v*alue**
**Crude models**			
FT4 (ng/dl)		−0.182 (−0.368–0.005)	0.057
TSH (mU/L)		0.00(−0.014–0.015)	0.969
TPOAb (IU/ml)		0.00 (0.00–0.00)	0.788
TPOAb	Positive	0.03 (−.15, .20)	0.775
	Negative	(ref)	
**Adjusted model 1#**			
FT4 (ng/dl)		−0.058 (−0.246–0.130)	0.544
TSH (mU/L)		0.005 (−0.010–0.019)	0.526
TPOAb (IU/ml)		0.00 (0.00–0.00)	0.822
TPOAb	Positive	0.03 (−.15, .20)	0.744
	Negative	(ref)	
**Adjusted model 2##**			
FT4 (ng/dl)		−0.043 (−0.232–0.146)	0.657
TSH (mU/L)		0.004 (−0.010–0.018)	0.592
TPOAb		0.00 (0.00–0.00)	0.785
TPOAb	Positive	0.03 (−.15, .20)	0.744
	Negative	(ref)	
**Exposures**		**Coefficient for Parity(95%CI)**	***P_*value**
**Crude mode**			
FT4(ng/dl)		−0.634 (−0.863 – − 0.405)	0.000*
TSH (mU/L)		−0.010 (− 0.027–0.008)	0.291
TPOAb (IU/ml)		0.00 (0.00–0.00)	0.891
TPOAb	Positive	− 0.2 (−.18, .14)	0.830
	Negative	(ref)	
**Adjusted model 1#**			
FT4 (ng/dl)		−0.103 (− 0.298–0.93)	0.304
TSH (mU/L)		0.005 (− 0.010–0.020)	0.528
TPOAb		0.00 (0.00–0.00)	0.988
TPOAb	Positive	− 0.02 (−.16, .11)	0.672
	Negative	(ref)	
**Adjusted model 2#**			
FT4(ng/dl)		−0.089 (− 0.283–0.106)	0.371
TSH (mU/L)		0.006 (− 0.009–0.021)	0.414
TPOAb (IU/ml)		0.00 (0.00–0.00)	0.968
TPOAb	Positive	− 0.02 (−.16, .11)	0.672
	Negative	(ref)	

*significant *p*-value < 0.05, # model adjusted for age, BMIand infertility status, ## model adjusted for age,education, BMI, fertility status,smoking, Systolic Blood Pressure (SBP), Diastolic Blood Pressure (DBP), Fasting blood Sugar (FBS

## Discussion

In the present study, we found that TPOAb positivity was not associated with infertility (male or female), abortion, time to pregnancy and parity. Our study revealed there was no association between infertility and other thyroid function tests with both male and female infertility, except fot Ft4; there was a positive association between serum concentration of Ft4 among male participants with infertility.

Thyroid antibodies may affect reproductive system with alteration in TSH/T4 levels directly but other mechanisms such as association with anti thyroglobolin, abnormal innate and humoral immunity, concurrent autoimmunity (i.e, endometriosis), vitamin D deficiency can ensue implantation failure in the absence of thyroid dysfunction [3].

Data on the association between thyroid autoimmunity and infertility is inconclusive and limited by limited number of comprehensive population based study [13, 20–22]. In a cross-sectional study on 11,565 Europian origin women, they found a negative association between TPOAb levels and parity; but not with the number of pregnancies and spontaneous abortion [13]. The difference in race and age distribution may partly explain different results.

In a case-control study in Iran by HABIB ZV et al. in 75 euthyroid women with unexplained infertility, serum anti-TPO antibodies were significantly higher in infertile euthyroid women comparing to healthy groups [20]. In another case-control study by Gupta J et al. in 50 infertile women, anti-thyroid antibodies were more prevalent in patients with infertility [21] and Manhas S et al. reported in 100 infertile female, Anti-TPO Ab was independently associated with infertility irrespective of thyroid hormones levels [22]. The positive findings of these case control studies may be due to selection biased that been mainly resulted from recruitment of cases from infertility centers. More over despite lack of normality assumption for TPOAb levels, they compared means for reporting significany. Furtheremorethe milder subjects may be spontaneously fertilized and not included in the study and this problem eliminates in a population-based study.

Our study is in agreement with study conducted by Soltanghoraee H et al.; they found no significant difference between median of TPOAb of infertile men/women and fertile ones (2.6 IU/ml vs 2.6 IU/ml in male, 2.8 IU/ml vs 2.6 IU/ml in female). Moreover they observed no association between thyroid autoimmunity and spontaneous abortion, similar to our results.

There are several studies investigate the prevalence of thyroid autoimmunity in women seeking infertility services. Marcos Abalovich et al. demonstrated no significant difference between TAI prevalence of infertile women with the control group(26.6% vs 14.5%) [23]. K. Poppe et al. conducted a study in 438 infertile women to investigate the prevalence of thyroid antibodies in infertile women with different causes included female or male cause and idiopathic. The thyroid test and thyroid autoimmunity association were evaluated in different groups of infertile females. The prevalence of infertility was 45, 38 and 17% in the female cause, male cause and idiopathic. TPOAb positivity was higher in all women of infertile couples but with no significant difference. In subgroup infertile women only in endometriosis TPOAb positivity showed significant difference compared with controls (18% vs 8%) [24].

In a study by Grassi G et al. in 149 infertile couples with the evaluation of the etiology of infertility and thyroid autoantibodies (Tg Ab, microsomal Ab), 20.1% had a detectable level of TgAb and /or M Ab and in euthyroid infertile women with thyroid autoantibodies, detected no difference either in age of subjects or duration of infertility [25].

There is a limited number of studies reported thyroid autoimmunity among male with infertility. Thyroid autoantibodies can be associated with sperm antibodies in infertile men [26, 27]. In a study by Harald Trummer et al. that been conducted among 305 infertile men; they reported no significant correlation between thyroid dysfunction and semen parameters, except for TPOAb positivity that was positively associated with pathozoospermia (6.7% vs 1.6%, *P* = .036) and asthenozoospermia (7.2%vs 1.6%, *P* = .049) [7]. However they have not reported the association of autoimmunity and thyroid dysfunction with male infertility despite having adequate sample size.

In the present study, we found a negative association between fT4 level in males (in both crude and adjusted models) with infertility. It has been shown that testosterone levels is decreased in hypothyroid men due to effect on hypothalamus-pituitary or alteration on prolactin secretion [28]. Indeed there is a significant decrease in male gonadal steroids suffered from hypothyroidism [29]. However there is no evidence to advice systematic screening for thyroid autoimmunity in infertile men without obvious clinical symptoms [30]. In the present study, we found a fall in fT4 level in both crude and adjusted models in males was associated with infertility although the TSH level showed no significant association that insufficient sample size could be the possible cause.

The current study has several limitations. We obtain data on infertility based on a self – reported questionnaire and observing their medical records if needed. Although there is a possibility of reducing the accuracy of self – reported questionnaire, however it may not mainly influenced our results as it an acceptable method in the population-based study for infertility [31]; moreover history of infertility that much affects the couple life that they may never forget it and Second, we used a single measurement of thyroid test in the present study, while it should be noticed that thyroid status may be varied by the time. Even though Somwaru et al. reported that subclinical hypothyroidism can be persistent in 56% at least for a 4-year follow-up [32]. Third, the number of men with confirmed male infertility was small and we had no adequate power for some comparison. Forth we have not assessd other parameters for assessment of thyroid autoimmunity such as anti- thyroglobulin.

The main strength of our study is its methodology; as it is one of the few population-based studies about thyroid autoimmunity on infertility, especially male infertility.

All immunoassay tests in present study were performed in the same laboratory by skilled laboratory technicians. Furthermore, we excluded those participants using thyroid medication or those with overt hypothyroidism - hyperthyroidism to eliminate the effect of treatment on TFTs.

## Conclusion

In conclusion, thyroid autoimmunity is not associated with infertility in both females and males. There is not sufficient data to recommend the infertile couple for assessment of thyoid autoimuninty or subclinical thyroid dysfunction. Further comprehensive population based studies with adequate sample size are highly suggested.

## Supplementary Information

**Additional file 1.** Reproductive questionnaire

## Data Availability

The datasets used and/or analysed during the current study available from the corresponding author on reasonable request.
