# Supplementary material for: The association between subclinical hypothyroidism and TPOAb positivity with infertility in a population-based study: Tehran thyroid study (TTS)
Source: BMC Endocr Disord. 2021 May 26;21:108. doi: 10.1186/s12902-021-00773-y (PMC8152029; doi:10.1186/s12902-021-00773-y)
Supplement: Supplementary file 1 — Additional file 1. Reproductive questionnaire [file 12902_2021_773_MOESM1_ESM.docx]

**Supplementary**

**The association between subclinical hypothyroidism and TPOAb positivity with infertility in a population-based study: Tehran Thyroid Study (TTS)**

**Reproductive questionnaire**

Batul Birjandi^1^, Fahimeh Ramezani Tehrani^2^, Atieh Amouzegar1, Maryam Tohidi^3^, Razieh Bidhendi Yarandi (PhD)^4,2,^ Feriedoun Azizi^1^

1. Endocrine Research Center, Research Institute for Endocrine Sciences, Shahid Beheshti University of Medical Sciences
2. Reproductive Endocrinology Research Center, Research Institute for Endocrine Sciences, Shahid Beheshti University of Medical Sciences, Tehran, Iran.
3. Prevention of Metabolic Disorders Research Center, Research Institute for Endocrine Sciences, Shahid Beheshti University, Tehran, Iran.
4. Department of Biostatistics, University of Social Welfare and Rehabilitation Sciences, Tehran, Iran.

**Corresponding author**

Atieh Amouzegar, MD.

Associate Professor of Internal Medicine and Endocrinology

Associated director Endocrine Research Center, Research Institute for Endocrine Sciences, Shahid Beheshti University of Medical Sciences, Tehran, Iran

P.O. Box: 19395-4763

Tehran, I.R. Iran

Phone: + 98 21 22432500

Fax: + 98 21 22402463

Email: Amouzegar@endocrine.ac.ir

**Age: Age of marriage (yr): Education:**

**BP: BMI: Smoking:**

**Contraceptive behaviors: Menstrual pattern:**

**Have you ever been pregnant?**

**yes No**

| **If Yes** | **If No** |
| --- | --- |
| number of parity:  number of birth:  number of abortion: | Have you ever had willingness for pregnancy?  Yes : No: |
| How long after your willingness for pregnancy, you got pregnant?  Month: Year: | How long after you have not been able to get pregnant?  Month: year: |
| If it has taken more than one year, would you visit any doctor for?  Yes: No: | If it has taken more than one year, would you visit any doctor for?  Yes: No: |
| What was the diagnosis if you visit a doctor:   - ovulatory factor: - Tubal factor: - Uterus factor: - Male factor: - unknown: | What was the diagnosis if you visit a doctor:   - ovulatory factor: - Tubal factor: - Uterus factor: - Male factor: - unknown: |
| What treatment were used for your infertility:   - Pharmacological agent: - Assisted reproductive technologies such as IUI,IVF - Surgical procedures: - Without any treatment: | What treatment have been used for your infertility:   - Pharmacological agent: - Assisted reproductive technologies such as IUI,IVF - Surgical procedures: - Without any treatment |
